# The UK COVID-19 Response: A Behavioural Irony?

**DOI:** 10.1017/err.2020.22

**Published:** 2020-04-02

**Authors:** Anne-Lise SIBONY

**Affiliations:** * Professor of EU Law, UCLouvain, Belgium; email: anne-lise.sibony@uclouvain.be.

The diversity of responses to the COVID-19 outbreak across countries both internationally and within the European Union (EU) is considerable and the lack of a coordinated response at the EU level is being criticised.^1^ Within this natural experiment involving different national policies, possibly the most strikingly distinct path is the one initially chosen by the UK and in which The Netherlands is persevering. Instead of trying to avoid contamination as much as possible though drastic measures such as early lockdown, the strategy is to encourage herd immunity.^2^ In the UK, this initial policy choice was presented as being based on both epidemiology and behavioural sciences.^3^ “Behavioural fatigue”, a little-known phrase not found in the most comprehensive textbook,^4^ suddenly rose to (probably short-lived) fame.^5^ The suggestion was that people would get tired of staying home so lockdown would be ineffective. In The Netherlands, the Prime Minister announced similarly relaxed rules about social distancing, and though he did not explicitly refer to any behavioural input, it is nonetheless highly likely that there was one.^6^ The initial moves of both of these governments met with scepticism and appeared shocking to many. The association between behavioural input in the policy decision and the decision to let the virus spread by refraining from ordering lockdown is unfortunate, but it is there. “How could a government rely merely on nudges in the face of grave danger?” observers legitimately asked.^7^ While no government relies merely on nudges (even The Netherlands has ordered schools and bars to close), this particular episode in the unfolding worldwide coronavirus saga gives behaviourally minded analysts pause. It is worth considering the proper place of behavioural insights in the difficult policy choices at hand.

## I. Difficult choices

Trying to eradicate the virus through comprehensive lockdown (suppression) has the advantage that it appears to work. As far as one can tell, it seems to have worked in China.^8^ It has the drawback that is it hugely costly for the economy and that the number of cases will still be large enough to overwhelm even well-resourced health systems.^9^ This means not only that many people will die, including overworked and exposed health professionals, but also that morally unpleasant choices will have to be made. In addition, it may be necessary to prolong lockdown for months or repeat it many times over if a new wave of infection occurs as soon as measures are relaxed, which is entirely likely in the absence of herd immunity.^10^ Mitigation, on the other hand, aims to create precisely such herd immunity. It consists in limiting isolation to groups most at risk of developing severe symptoms and letting the virus infect large sections of the remaining population. The social and economic disruption is more limited with this strategy, but it has the drawback that people most at risk might be misidentified, thus causing deaths that could have been avoided with more drastic measures. Importantly, it is also irreversible: after the virus has spread widely, isolation will not be as effective as it would have been in the early stages. As *The Economist* sums up, “The bitter truth is that mitigation costs too many lives and suppression may be economically unsustainable”.^11^ Indeed, it has been estimated when then choice was made to go for herd immunity that 80% of the UK population would be infected, plausibly resulting in half a million deaths.^12^ On the other hand, the French National Statistical Institute has calculated that production dropped by 35% because of the lockdown,^13^ which means that each month of this policy costs three points of annual GDP.^14^

Choosing the right policy in these circumstances is challenging for many reasons, starting with a multifaceted knowledge problem: we have only partial knowledge of the virus, of exactly how it spreads and how immunity builds up or what existing drugs could help. There is neither enough data^15^ nor enough time to conduct serious cost–benefit analyses (leaving aside ethical concerns about the utilitarian morality underpinning such analyses and the reluctance to put a price tag on lives).^16^ Even risk analysis seems to have been neglected. In addition to all that we do not know, there are logistical constraints pertaining to the production of protective equipment, sanitising gel or ventilators. There are conflicting values, and some trade-offs are becoming increasingly salient, such as balancing health and privacy.^17^ Finally, the epidemic does not shut down politics: just recall how France held the first round of municipal elections during the first week of lockdown after the President gave in to the opposition of the President of the Senate,^18^ or how Prime Minister Orbán is using the virus to advance his political agenda of an ever-tighter grip over Hungarian institutions.^19^ In addition to scientific uncertainties, painful trade-offs and economic and logistical constraints, policy-makers also have to reckon with behavioural factors.

## II. Behavioural dimensions

Quite obviously, citizens’ behaviour affects how the virus spreads. Getting people to behave in certain ways can literally save lives. In other words, the stakes have never been so high when it comes to incorporating behavioural insights into policy design. Yet, getting things right is a tall order as the behaviour changes needed are far-reaching, but also because governments are under intense pressure to make decisions fast, under several veils of uncertainty and in a context characterised by both rational fear of experts and irrational optimism of citizens, many of whom feel they are not personally at risk. All of these circumstances offer fertile ground for biases and errors.

### 1. Promoting safe behaviour: a natural turf for behavioural insights

In the absence of treatment or a vaccine, the only way to slow down the progression of the virus is changing behaviour. Such change needs to occur in connection with deliberate behaviour, such as going out or washing one’s hands; with conscious but habitual behaviour, such as greeting people or standing close to them; and with mostly unconscious behaviour, such as touching one’s face.^20^ Initiating rapid behavioural change on a massive scale is not the usual business of governments, nor is the regulation of micro-social interaction, in principle, the purpose of law. Yet, in the absence of a vaccine and amid controversies on the efficacy of available drugs, “non-pharmaceutical interventions” are initially the single most important tools to try and protect populations’ health (leaving aside the indirect health risks generated by loss of income over time).^21^ Things are rapidly evolving: some governments have allowed use of existing antiviral drugs in hospitals, a French Court has recently ordered health authorities to stock up on such drugs^22^ and a vaccine may be within reach.^23^ Yet, it remains that behavioural factors are crucial, and there is little doubt that this dimension has a place in policy design.

Broadly speaking, the context is favourable to such an approach, as governments in many parts of the world have been increasingly turning to behavioural expertise over the past decade.^24^ The 2019 Nobel Prize in Economic Sciences has been given to researchers who have demonstrated the potency of seemingly modest interventions to improve health and well-being using randomised controlled trials, the same experimental methodology as is recommended to test and adapt behavioural interventions.^25^ In addition, and despite controversies among academics about libertarian paternalism, preliminary empirical evidence suggests that Europeans like nudges when they approve of the underlying policy aim and if governments adhere to the basic rules of “good governance of nudging”^26^ (data are as yet lacking as to whether these findings also apply to UK politicians).^27^ Against this backdrop, why did the initial announcement of behaviourally inspired interventions run up against strong criticism in the present context?^28^

### 2. Objections to the UK response: junk behavioural science

To clarify, the objection was not directed against adopting behaviourally inspired measures per se; it was about *not* adopting (at the time) strict social distancing measures. The critique was also not directed at the specific behavioural recommendations that were issued, such as advice on how to stop touching one’s face^29^ or how best to nudges people to wash their hands effectively.^30^ Such recommendations cost very little and can go some way in the right direction; certainly, no one objects to them.

The objection was that behavioural arguments that were presented as supporting the decision to wait rather than adopt drastic measures were ill-founded. The fear that “behavioural fatigue” – as the UK government called it – might kick in and undermine the effectiveness of a lockdown as people would start violating the recommendation to stay home may be intuitively plausible but, behavioural scientists said, is not a documented behavioural phenomenon.^31^ Not adopting a potentially life-saving lockdown based on the mere intuition that people may get tired of it simply is not good enough. It is not evidence-based behavioural policy-making.

### 3. Multiple behavioural phenomena

Importantly, it is not clear why behavioural fatigue was singled out given that other, better-documented behavioural phenomena might – with equally unknown probability and distribution – be at work and either fuel or counteract it.^32^ Besides behavioural fatigue, non-compliance with social distancing measures could be the result of the optimism bias, which can lead people to believe they are less likely to acquire a disease.^33^ Another potential cause of non-compliance with stay-at-home exhortations is reactance: that is, an appetite for doing the opposite of what we are told when we feel our freedom of choice is being limited. Clearly, the baseline level of reactance in a population is a cultural trait. Anecdotal evidence suggests, for example, that it is lower in Belgium than in France, where Parisians flocked en masse to their vacation homes the day before lockdown, elbowing their way through crowded train stations to packed TGVs. Increases or decreases in reactance from the baseline level are plausibly influenced by how politicians and other public figures behave.^34^ In that regard, it is striking how Boris Johnson, Donald Trump or Emmanuel Macron undermined the official message of social distancing by publicly shaking hands, attending meetings or visiting factories, while Angela Merkel and Sophie Wilmès, the Belgian Prime Minister, led by example from the beginning. Reactance can be mitigated by targeting different subgroups,^35^ such as young people, which some governments have done through social media campaigns highlighting that caring for elders is cool. Reactance can also be mitigated by involving citizens in policy development.^36^ While this might *prima facie* seem difficult in an emergency situation, it is also true that disastrous communication causes deaths, and taking a little bit of time to help officials grasp the mood(s) in the population might in fact be time well spent.

On the other hand, behavioural fatigue might be offset by fear of the disease. Fear is known to be a powerful motivator, though here, again, we lack data that are directly relevant to the current context.^37^ Importantly, sound behavioural arguments in favour of enforcing lockdown and social distancing through law (rather than mere recommendations) seem to have received too little consideration. First, we all hold conflicting voices in our minds: the voice of quick, intuitive emotional decisions wants us to go and see friends and the voice of reasoned decision that weighs pros and cons rationally and argumentatively will suggest that this is not reasonable. And we know which one usually wins (this is known as dual process theory).^38^ This is why it is a good idea to take decisions as to whether or not to see friends off people’s shoulders.^39^ Once “Stay At Home” is the law rather than a recommendation, voluntary compliance could be the result of citizens recognising the expressive function of the law.^40^ This may be a more reliable mechanism than reliance on social norms where the social norm is not well established.^41^

In other words, there are behavioural phenomena that go in different directions and we simply do not know enough about these behavioural trade-offs.^42^ Quite possibly, individual differences are large and the net effect of these phenomena depends at least in part on demographics. By way of illustration, a Belgian survey suggests that 25% of the population and as much as 44% of 18–21-year-olds do not observe the social distancing measures.^43^ In truth, the behavioural and social sciences help formulate many hypotheses of what psychological and social levers might drive risky behaviour but no hard evidence that is directly policy-relevant. Making “behavioural fatigue” uniquely salient to justify a minimalist policy simply seems random. Why, then, did “behavioural fatigue” have the honour of featuring as policy justification?

### 4. A behavioural explanation?

Could the focus on an obscure and undocumented behavioural phenomenon (which may well exist nonetheless) be an instance of group reinforcement in the decisional process? The 2018 Behavioural Insights Team (BIT) Report *Behavioural Government* (the UK Nudge Unit’s manual for de-biasing governments) explains that this form of groupthink happens when people self-censor and conform to the group majority view.^44^ Did herd immunity emerge as the best candidate for consensus among medical experts? Might it have received a subtle cultural push (Brits keep calm and carry on)? Was the time pressure so strong that whatever would appear to bring the voice of behavioural insights in line with other disciplines represented in the expert group got picked out? The fact that there was no response to the open letter from nonplussed behavioural scientists does suggest that this was not the best that the behavioural approach can offer to policy-making in general or to COVID-19 response in particular. The UK Nudge Unit may have deserved a taste of its own medicine, but it should be recognised that evidenced-based policy-making is exceptionally difficult to deliver when there are precious few data and no time to gather evidence.

Another explanation for the initial choice of herd immunity over early lockdown is the framing effect. In a famous experiment that uniquely resonates with the current context, Kahneman and Tversky showed that decisions about risks are influenced by how the choice is framed.^45^ In this experiment, subjects were told that a community faced an unusual Asian disease that was expected to kill 600 people. To combat the disease, the first group could choose between two options: Option A consisted in a treatment that would ensure 200 people would be saved and Option B in a treatment that had a 33% chance of saving all 600 but a 67% chance of saving none. Option A was the clear winner (chosen by 72% of subjects). A second group was presented with the same choice but framed differently. This group had to choose between Option C, which would result in only 400 people dying, and Option D, characterised by a 33% chance that nobody would perish and a 67% chance that all 600 would die. This time 72% favoured Option D. This is puzzling because Option D is equivalent to Option B. In other words, when the same choice (between a certain option and a probabilistic one with identical expected utility) was framed differently, people’s risk preferences were reversed. In the positive framing (number of lives saved in Options A and B), subjects preferred certainty, but in the negative framing (number of deaths in Options C and D), they were willing to take risks. It has been shown that politicians are not immune to framing effects: they, too, are willing to take more risks to avoid deaths than to save lives (even though numbers make the options equivalent).^46^ In the current context, we constantly hear and read about the death toll of the virus: the framing is clearly negative. Prospect theory predicts that this will lead to more risk-taking. Alas, the initial decisions to delay strict social distancing – whether dressed up or not in behavioural arguments – may illustrate this prediction.

We are living through a natural experiment: nation-states worldwide and throughout Europe have developed different policy responses to the COVID-19 pandemic, ranging from locked-up French and Spaniards to free-range Swedes. Within the coming months, evidence will show which strategies were most effective, cost-efficient and socially accepted. But this is also an example of how governments can misuse behavioural arguments and tarnish a reputation for sound evidence-based policy-making. Those in Europe who have applied behavioural insights most effectively may not be the ones who boast about it.

## Footnotes

1 A Alemanno, “Europe Doesn’t Have to Be So Helpless in This Crisis”, *The Guardian*, 26 March 2020 <https://www.theguardian.com/world/2020/mar/26/europe-doesnt-have-to-be-so-helpless-in-this-crisis>. See also the petition calling for a coordinated EU response: <https://thegoodlobby.eu/campaigns/covid19-europe-can-fight-it>.

2 While it is true that Boris Johnson’s statement of 10 March 2020 about “taking the virus on the chin and let it move through the population” has been taken out of context (the Prime Minister said this was “one theory” and then outlined his preferred one), it remains true that the UK has taken more limited social isolation measures than other countries. Full transcript of the Prime Minister’s televised interview available at: <https://fullfact.org/health/boris-johnson-coronavirus-this-morning>. Similarly, in The Netherlands, Prime Minister Rutte announced the Dutch strategy of building herd immunity on 16 March 2020 in a televised speech (the first of a Prime Minister since World War II) <https://www.rijksoverheid.nl/documenten/toespraken/2020/03/16/tv-toespraak-van-minister-president-mark-rutte>. The Prime Minister does not use the phrase “herd immunity”, but an interesting distinction between “maximum *control*” (the label for the chosen strategy of no lockdown) and lockdown.

3 See S Boseley, “Herd Immunity: Will the UK’s Coronavirus Strategy Work?” *The Guardian*, 13 March 2020, quoting David Halpern, head of the Behavioural Insights Team <https://www.theguardian.com/world/2020/mar/13/herd-immunity-will-the-uks-coronavirus-strategy-work>; Institute for Government (UK) “Explainer” on the “Nudge Unit” <https://www.instituteforgovernment.org.uk/explainers/nudge-unit>. The Scientific Advisory Group on Emergencies (SAGE) advisors to the government include “epidemiologists, clinicians, virologists, mathematical and statistical modellers, molecular biologists and social and behavioral scientists”. C Cooper and A Furlong, “Going Viral: Boris Johnson Grapples to Control Coronavirus Message”, *Politico*, 16 March 2020 <https://www.politico.eu/article/going-viral-british-prime-minister-boris-johnson-grapples-to-control-coronavirus-covid19-message>.

4

E Zamir and D Teichman , Behavioural Law and Economics (Oxford, Oxford University Press 2018 ).

5 N Chater, “People Won’t Get ‘Tired’ of Social Distancing – The Government Is Wrong to Suggest Otherwise”, *The Guardian*, 16 March 2020 <https://www.theguardian.com/commentisfree/2020/mar/16/social-distancing-coronavirus-stay-home-government>.

6 This is not only a presumption based on the fact that the Dutch government is among the most behaviourally aware in the EU, but more specifically because the Dutch National Institute for Public Health and the Environment, whose role it is to advise the government, enjoys in-house expertise on behavioural change among both senior and junior staff.

7 The term ‘nudge’ has been popularised by RH Thaler and CR Sunstein , Nudge: Improving Decisions about Health, Wealth, and Happiness (New Haven, CT, Yale University Press 2008 ), who define it as “any aspect of the choice architecture that alters people’s behaviour in a predictable way without forbidding any options or significantly changing their economic incentives”. For helpful clarifications and a more precise definition, see PG Hansen , “The Definition of Nudge and Libertarian Paternalism: Does the Hand Fit the Glove?” (2016) 7 European Journal of Risk Regulation 155 ; and A Tor , “The Critical and Problematic Role of Bounded Rationality in Nudging” in K Mathis and A Tor (eds), Nudging – Possibilities, Limitations and Applications in European Law and Economics (Berlin, Springer 2016) pp 3–10 ; F Esposito , “Conceptual Foundations of the Behavioural Analysis of Consumer Law in Europe” in H-W Micklitz , A-L Sibony and F Esposito (eds), Research Methods in Consumer Law (Cheltenham, Edward Elgar 2018) pp 38–76 .

8
ibid.

9
ibid, citing the Imperial College report, which predicted that peak demand for intensive care in the UK would still be eight times the surge capacity of Britain’s National Health Service: Imperial College COVID-19 Response Team, “Impact of Non-Pharmaceutical Interventions (NPIs) to Reduce COVID-19 Mortality and Healthcare Demand”, 16 March 2020 <https://doi.org/10.25561/77482>.

10 European Centre for Disease Prevention and Control, *Guide to Revision of National Pandemic Influenza Preparedness Plans – Lessons Learned from the 2009 A(H1N1) Pandemic* (Stockholm, ECDC 2017) p 15.

11 “Paying to Stop the Pandemic”, *The Economist*, 19 March 2020 <https://www.economist.com/leaders/2020/03/19/paying-to-stop-the-pandemic>.

12 Figure published by *The Guardian*, citing “a senior NHS official”. D Campbell, “UK Coronavirus Crisis ‘to Last Until Spring 2021 and Could See 7.9m Hospitalised’”, *The Guardian*, 15 March 2020 <https://www.theguardian.com/world/2020/mar/15/uk-coronavirus-crisis-to-last-until-spring-2021-and-could-see-79m-hospitalised>.

13 INSEE, “Point de conjoncture”, 26 March 2020 <https://www.insee.fr/fr/information/4471804>.

14 G de Calignon, “Coronavirus: un mois de confinement représente une perte de 3 points de PIB annuel, selon l’INSEE”, *Les Echos*, 26 March 2020 <https://www.lesechos.fr/economie-france/conjoncture/coronavirus-un-mois-de-confinement-represente-une-perte-de-3-points-de-pib-annuel-selon-linsee-1189241>.

15 On the dismal statistics, see D Bessis, “Coronavirus: The Key Numbers We Must Find Out”, *Medium*, 26 March 2020 <https://medium.com/@davidbessis/coronavirus-the-core-metrics-we-should-be-looking-at-2ca09a3dc4b1>.

16 For a discussion and an explanation of why in the USA a life is valued at US$9 million, see C Sunstein , The Cost Benefit Revolution (Cambridge, MA, MIT Press 2019) p 39 .

17 Privacy laws should be “paused” as part of cutting red tape to help fight the coronavirus, write behavioural economists Sendhil Mullainathan and Richard H Thaler in “To Fight the Coronavirus, Cut the Red Tape”, *The New York Times*, 24 March 2020 <https://www.nytimes.com/2020/03/24/business/coronavirus-medical-supplies-regulations.html>. A Reenda, “Will Privacy Be One of the Victims of COVID-19”, *CEPS*, 23 March 2020 <https://www.ceps.eu/will-privacy-be-one-of-the-victims-of-covid-19/?mc_cid=d9dce71bc5&mc_eid=d5d54749b6>.

18 M Mourgue, “Coronavirus: Larcher s’est opposé auprès de Macron à un report des municipales”, *Le Figaro*, 12 March 2020 <https://www.lefigaro.fr/elections/municipales/coronavirus-emmanuel-macron-et-gerard-larcher-vont-s-appeler-pour-evoquer-les-municipales-20200312>.

19 J Dempsey, “Orbán Exploits Coronavirus Pandemic to Destroy Hungary’s Democracy”, *Carnegie Europe*, 31 March 2020 <https://carnegieeurope.eu/strategiceurope/81410>.

20 Such unconscious behaviours are hardest to tackle: <https://www.bi.team/blogs/how-to-stop-touching-our-faces-in-the-wake-of-the-coronavirus>.

21 Imperial College COVID-19 Response Team, “Impact of Non-Pharmaceutical Interventions (NPIs) to Reduce COVID-19 Mortality and Healthcare Demand”, supra, note 9.

22 Administrative Court of Guadeloupe, 28 March 2020, Syndicat UGTG, no. 2000295.

23 I Efrati and C Levinson, “Israeli Research Center to Announce It Developed Coronavirus Vaccine, Sources Say”, *Haaretz*, 18 March 2020 <https://www.haaretz.com/israel-news/.premium-coronavirus-vaccine-israel-biological-research-institute-develope-1.8665074>.

24 The UK pioneered the move with the creation of the Behavioural Insights Team in 2010. Since then, many governments around the developed world have explored the potential of behavioural interventions. For a survey, see OECD, “Behavioural Insights and Public Policy: Lessons from Around the World”, 2017 <https://www.oecd.org/gov/regulatory-policy/behavioural-insights-and-public-policy-9789264270480-en.htm>. Highlighting the promises of behavioural policy-making in developing countries, see World Bank, “World Development Report 2015: Mind, Society, and Behavior” <https://www.worldbank.org/en/publication/wdr2015>. Within the European Commission, behavioural expertise is dispensed by the Joint Research Center as well by a growing number of smaller teams within the directorate-general. For an EU focus, see JS Lourenço et al, “Behavioural Insights Applied to Policy: European Report 2016” <https://publications.jrc.ec.europa.eu/repository/bitstream/JRC100146/kjna27726enn_new.pdf>.

25 The 2019 Nobel Prize in Economic Sciences was awarded jointly to Abhijit Banerjee, Esther Duflo and Michael Kremer for their experimental approach to alleviating global poverty: <https://www.nobelprize.org/prizes/economic-sciences/2019/summary>. On the use of randomised controlled trials for behavioural interventions, see Behavioural Insights Team with B Goldacre and D Torgerson, “Test, Learn, Adapt: Developing Public Policy with Randomised Controlled Trials”, Cabinet Office The Behavioural Insights Team, 2013 <https://www.bi.team/publications/test-learn-adapt-developing-public-policy-with-randomised-controlled-trials>.

26

L Reisch , C Sunstein and W Gwozdz , “Viewpoint: Beyond Carrots and Sticks: Europeans Support Health Nudges” (2017) 69 Food Policy 1 ; L Reisch and C Sunstein , Trusting Nudges: Toward a Bill of Rights for Nudging (Abingdon, Routledge 2019 ) ch 6.

27 The shadow health secretary, Jon Ashworth, is reported to have asked for reassurance that the government approach was not based too much on behavioural science. H Stewart and M Busby, “Coronavirus: Science Chief Defends UK Plan from Criticism”, *The Guardian*, 13 March 2020 <https://www.theguardian.com/world/2020/mar/13/coronavirus-science-chief-defends-uk-measures-criticism-herd-immunity>.

28 T Yates, “Why Is the Government Relying on Nudge Theory to Fight Coronavirus?” *The Guardian*, 13 March 2020 <https://www.theguardian.com/commentisfree/2020/mar/13/why-is-the-government-relying-on-nudge-theory-to-tackle-coronavirus>.

29 M Hallsworth, “How to Stop Touching Our Faces in the Wake of the Coronavirus”, *BIT Blog*, 5 March 2020 <https://www.bi.team/blogs/how-to-stop-touching-our-faces-in-the-wake-of-the-coronavirus>.

30 M Hallsworth, “Handwashing Can Stop a Virus – So Why Don’t We Do It?” *Behavioural Scientist*, 4 March 2020 <https://behavioralscientist.org/handwashing-can-stop-a-virus-so-why-dont-we-do-it-coronavirus-covid-19>; and M Egan et al, “Bright Infographics & Minimal Text Make Handwashing Posters Most Effective – Result from an Online Experiment”, *BIT Blog*, 23 March 2020 <https://www.bi.team/blogs/bright-infographics-and-minimal-text-make-handwashing-posters-most-effective>.

31 U Hahn et al, “Why a Group of Behavioural Scientists Penned an Open Letter to the U.K. Government Questioning Its Coronavirus Response”, *Behavioural Scientist*, 16 March 2020 <https://behavioralscientist.org/why-a-group-of-behavioural-scientists-penned-an-open-letter-to-the-uk-government-questioning-its-coronavirus-response-covid-19-social-distancing>.

32 To date, the most comprehensive outlines of relevant behavioural phenomena are PD Lunn et al, “Using Behavioral Science to Help Fight the Coronavirus” (2020) 3 Journal of Behavioral Public Administration <https://doi.org/10.30636/jbpa.31.147> and JJ Van Bavel et al, “Using Social and Behavioural Science to Support COVID-19 Pandemic Response”, manuscript published online 24 March 2020 <https://psyarxiv.com/y38m9> (hereafter “Van Bavel”).

33 E Zamir and D Teichman, *Behavioural Law and Economics*, supra, note 4, 61; Van Bavel, text at n. 8.

34 S Bhanot, “Why Are People Ignoring Expert Warnings? – Psychological Reactance”, *Behavioural Scientist*, 20 March 2020 <https://behavioralscientist.org/why-are-people-ignoring-expert-warnings-psychological-reactance-coronavirus-covid-19>. An anecdotal example is a clandestine café in Belgium: “Dour: un café clandestin installé dans une arrière-salle”, *La Libre Belgique*, 29 March 2020 <https://www.lalibre.be/regions/hainaut/dour-un-cafe-clandestin-installe-dans-une-arriere-salle-5e80ae52d8ad58163186b3fb>.

35

P John , How Far to Nudge? (Cheltenham, Edward Elgar 2018) p 29 .

36
ibid, p 125.

37 D DeSteno, “How Fear Distorts Our Thinking about the Coronavirus”, *The New York Times*, 20 February 2020 <https://www.nytimes.com/2020/02/11/opinion/international-world/coronavirus-fear.html>.

38

D Kahneman , Thinking, Fast and Slow (New York, Farrar, Straus and Giroux 2011 ).

39 A Buttenhein, “Tradeoffs”, 12 March 2020 <https://tradeoffs.org/2020/03/12/cc-buttenheim>.

40 The hashtag #COVIDIOTS on Twitter illustrates naming and shaming to privately enforce the new social norms.

41 On different social norms regarding whether to wear a face mask, see T Wong, “Coronavirus: Why Some Countries Wear Face Masks and Others Don’t”, *BBC*, 31 March 2020 <https://www.bbc.com/news/world-52015486>.

42 On behavioural trade-offs, see Y Feldman and O Lobel , “Behavioral Tradeoffs: Beyond the Land of Nudges Spans the World of Law and Psychology” in A Alemanno and A-L Sibony , Nudge and the Law: A European Perspective (Oxford, Hart Publishing 2015) pp 301–24.

43 “Coronavirus: 44% des jeunes de 18 à 21 ans ne respecteraient pas les mesures de confinement”, *RTBF*, 25 March 2020 <https://www.rtbf.be/info/societe/detail_coronavirus-le-confinement-une-tannee-chez-44-des-jeunes-de-18-a-21-ans-selon-test-achat?id=10467310>.

44 M Hallsworth et al, “Behavioural Government”, The Behavioural Insights Team, 2018 <https://www.bi.team/publications/behavioural-government> (hereafter *Behavioural Government*).

45

A Tversky and D Kahneman , “The Framing of Decisions and the Psychology of Choice” (1981) 211 Science, 453 745568310.1126/science.7455683.

46
*Behavioural Government*, p 8.

